# The influence of social and spatial processes on the epidemiology of environmentally transmitted pathogens in wildlife: implications for management

**DOI:** 10.1098/rstb.2022.0532

**Published:** 2024-09-04

**Authors:** Aakash Pandey, Chris Wojan, Abigail Feuka, Meggan E. Craft, Kezia Manlove, Kim M. Pepin

**Affiliations:** ^1^ Department of Fisheries and Wildlife, Michigan State University, East Lansing, MI 48824, USA; ^2^ Department of Ecology, Evolution, and Behavior, University of Minnesota, Saint Paul, MN 55108, USA; ^3^ National Wildlife Research Center, USDA-APHIS, Fort Collins, CO 80521, USA; ^4^ Department of Wildland Resources and Ecology Center, Utah State University, 5200 Old Main Hill, Logan, UT 84322, USA

**Keywords:** social structure, spatial structure, host movement, epidemiology, environmental transmission, pathogen dynamics

## Abstract

Social and spatial structures of host populations play important roles in pathogen transmission. For environmentally transmitted pathogens, the host space use interacts with both the host social structure and the pathogen’s environmental persistence (which determines the time-lag across which two hosts can transmit). Together, these factors shape the epidemiological dynamics of environmentally transmitted pathogens. While the importance of both social and spatial structures and environmental pathogen persistence has long been recognized in epidemiology, they are often considered separately. A better understanding of how these factors interact to determine disease dynamics is required for developing robust surveillance and management strategies. Here, we use a simple agent-based model where we vary host mobility (spatial), host gregariousness (social) and pathogen decay (environmental persistence), each from low to high levels to uncover how they affect epidemiological dynamics. By comparing epidemic peak, time to epidemic peak and final epidemic size, we show that longer infectious periods, higher group mobility, larger group size and longer pathogen persistence lead to larger, faster growing outbreaks, and explore how these processes interact to determine epidemiological outcomes such as the epidemic peak and the final epidemic size. We identify general principles that can be used for planning surveillance and control for wildlife host–pathogen systems with environmental transmission across a range of spatial behaviour, social structure and pathogen decay rates.

This article is part of the theme issue ‘The spatial–social interface: a theoretical and empirical integration’.

## 1. Introduction

Pathogen transmission is intricately linked to the spatial and social structures of host populations, making disease an important mechanism by which the spatial–social interface can directly influence host fitness. Environmental pathogens, unlike directly transmitted or vector-borne pathogens, are transmitted through shared locations in space, making their dynamics particularly sensitive to not only the social configuration but also the spatial distribution and movement patterns of host populations. Different hosts must visit the same site at time-delays shorter than the pathogen’s environmental persistence time in order for transmission to occur, introducing a layer of complexity that is crucial to understanding the disease spread. While numerous studies have individually examined the roles of spatial dynamics [1–4], social structures [5–8] and environmental persistence of pathogens [9–13] in disease dynamics, research that untangles the relationships among these processes is scarce. A joint analysis of how environmental persistence, host social structure and host space use affect disease dynamics will provide insights about which host social behaviours, host movement motifs or pathogen traits are most critical for disease transmission.

Host space use and movement patterns can significantly influence pathogen exposure [4,14–16]. For example, land cover types and rodent population densities have been linked to the incidence of hantavirus pulmonary syndrome in humans [17,18], and spatial structure of the environment has been linked to aggregation patterns and subsequently structured disease transmission patterns in communally roosting bats, both theoretically [19] and empirically [20].

Distance and the frequency of movement impact the likelihood that hosts encounter pathogen-laden areas, also directly affecting transmission rates [21,22]. Host mobility and pathogen life history attributes—including duration of infection and environmental persistence—can interact to shape epidemiological outcomes that require further exploration.

The social structure of host populations plays a pivotal role in the transmission of infectious diseases [23]. Social phenotypes like gregariousness or group-size preference can either amplify or mitigate pathogen spread, depending on various factors such as group size, social hierarchy and interaction patterns [8,24–26]. For example, in wild male chimpanzee communities, social interactions and resulting network structure influence the spread of respiratory diseases, with more socially integrated individuals facing a higher risk of infection [27]. Within- and between-group contact patterns can also influence disease transmission. High within-group contact rates can lead to rapid within-group disease transmission but can facilitate pathogen fade-out when between-group contact rates are low [28,29]. Additionally, within- and between-group contacts can both be influenced by the spatial scale at which requisite resources are distributed over space. For example, between-group contact in wild pigs depends on the spatial distribution of group centroids, with most contacts occurring between groups with centroids less than 2 km apart [29]. The distance between group centroids is itself influenced by the distribution of a spatial resource, baiting sites, across the landscape, thereby influencing the spatial spread of the disease including African swine fever [29,30]. The multifaceted interplay of social behaviours, spatial extent and pathogen transmission influences epidemiological dynamics in ways that require further exploration.

Many pathogens can be transmitted from environmental ‘reservoirs’, with transmission rates influenced by time since environmental deposition [31]. How long a pathogen remains viable in the environment directly affects the probabilities and dosages of host exposure. For example, the longer persistence of spores from the protozoan parasite *Ophryocystis elektroscirrha* leads to a higher infection prevalence in monarch butterflies [32]; and factors like substrate, desiccation, temperature and sunlight that influence the environmental persistence of *Mycobacterium bovis* also alter the risk of *M. bovis* infection in cattle, badgers and deer [33,34]. Overlaying variable environmental persistence of different pathogens on heterogeneous patterns of host mobility and sociality can lead to varied epidemiological dynamics in humans and animals [4].

While the importance of considering interactions of spatial and social structures for predicting disease dynamics has been recognized [30,35], implications of these interactions for disease management are often poorly understood. There are few guidelines for predicting how disease dynamics of environmentally transmitted pathogens might be affected by different host spatial and social processes, making it difficult to identify the most appropriate surveillance designs or management interventions. Moreover, the interplay between the host’s spatial and social phenotypes and the pathogen’s environmental persistence can lead to complex epidemiological outcomes that may not be predictable from the study of each factor in isolation. Therefore, an integrated approach that combines host spatial behaviour, host social structure and environmental persistence is crucial for predicting and managing disease spread.

Herein, we address this gap by employing an agent-based stochastic susceptible–infected–recovered (SIR) model. We systematically vary key parameters: movement distance/mobility (spatial structure), host gregariousness (social structure) and environmental persistence time (pathogen decay) to elucidate the combined effects of these factors on the epidemiological dynamics of a range of (hypothetical) environmentally transmitted pathogens causing short and long infectious periods within a host. Our approach jointly accounts for host social structure, host spatial ecology and pathogen environmental persistence to reflect the complexities of real-world disease transmission and identify general principles for the management of wildlife disease.

## 2. Model description

**Figure 1.**
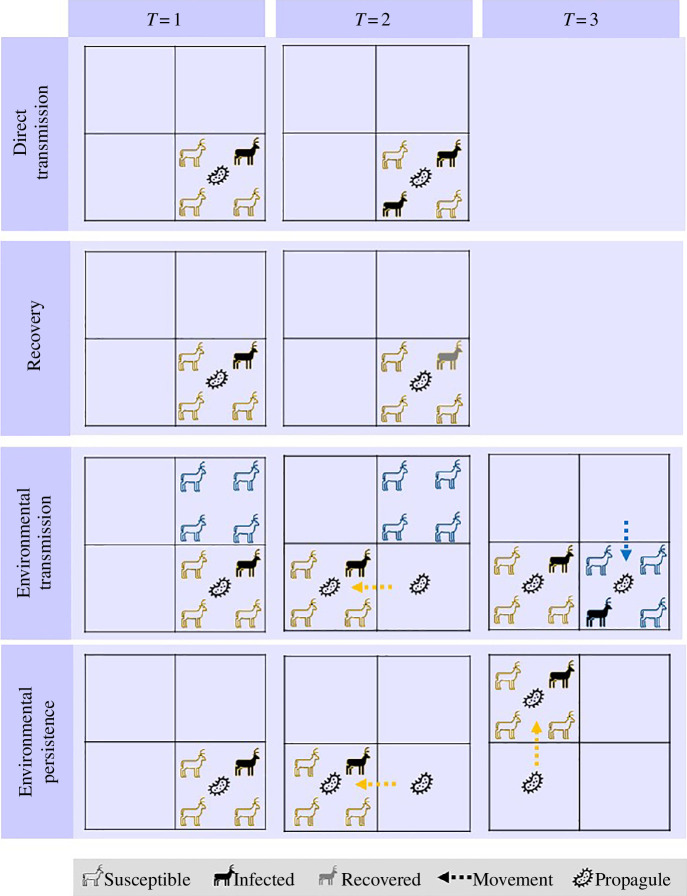
Processes captured in the agent-based stochastic SIR model, simplified to two groups of individuals (yellow and blue). Direct transmission occurs within groups with probability $\beta_{d}$ . Individual recovery occurs at rate γ after infection. Environmental transmission occurs when a group with an infected individual occupies a cell, leaves behind pathogen propagules through shedding, another group with susceptible individuals occupies that cell and can become infected with probability $\beta_{e}$ .

We developed a spatially explicit, agent-based, stochastic SIR model on a 20 × 20 lattice (figure 1), which considered both direct and environmental transmission. Individual ‘agents’ were transitioned from susceptible (S) to infected (I) to recovered (R) states according to a series of Bernoulli draws, generating approximately exponentially decaying waiting times within states. At each time step, we first updated disease transmission by allowing S individuals to transition to I owing to both direct and environmental exposure routes. Next, environmental grid cells became contaminated if infectious individuals were present and remained contaminated for a period determined by the persistence of the pathogen. This was followed by recovery (I to R transition), which occurred at a constant per capita rate, γ. Finally, group positions were updated as described below. The simulation ran at discrete, daily time steps until no infected individuals were present. For simplicity, we ignored demographic turnover and disease-induced mortality. To capture variable infection timelines, host social and spatial processes, and environmental persistence, we varied the infectious period (long, short), group size (large, medium, small), group mobility (high, low) and environmental persistence of the pathogen (long, short) (table 1). We selected parameter ranges that provided a dichotomy between the epidemiological traits we were interested in. For each scenario, we performed 1000 simulations and measured the epidemic peak, time to epidemic peak, final epidemic size (proportion of total population ever infected by the end of the epidemic), and the relative contribution of environmental and direct transmission to the final epidemic size.

**Table 1.** Definitions, units and ranges of epidemiological parameters used in the SIR model.

parameter	description	units	range
group size	number of individuals in group	individuals	small (5), medium (10), large (20)
infectious period	length of infection, function of recovery rate	days	short (10), long (30)
recovery probability	probability of surviving infection	daily individual probability	fast $\left(1-e^{-\frac{1}{10}}\right)$ slow $\left(1-e^{-\frac{1}{30}}\right)$
direct transmission	probability of a susceptible individual becoming infected via direct contact with another individual	daily individual probability	0.05
environmental transmission	probability of a susceptible individual becoming infected via contact with an environmental propagule	daily individual probability	0.03
environmental persistence	number of days pathogen survives in the environment as propagules	days	short (3), long (30)
mobility	probability of a group moving grid cells (*x*, *y*) daily	daily group probability	high: 0.88 chance of moving to an adjacent cell low: 0.36 chance of moving to an adjacent cell

### (a) Social structure and dispersal

We explored three different group sizes of 20, 10 and 5 individuals each. For each scenario, the total number of groups was fixed to 25. We checked the sensitivity of our results to changing population sizes for diseases with short infectious periods by running an alternative set of simulations in which the population was held constant at 200 individuals and the number of group numbers was varied (i.e. 40 groups of size 5, 20 groups of size 10, and 10 groups of size 20). We assigned the initial group locations within the lattice randomly. At each time step, each group either stayed in the same grid location or moved to a neighbouring cell. Movements were determined by sampling from the eight neighbouring grid locations (Moore neighbourhood) and the current location. This meant that each group moved in unison via uncorrelated, unbiased simple random walks within the lattice with reflective barriers at the edges [36] (although we checked the sensitivity of our results to edge effects by running an alternative set of simulations on a torus). We varied group mobility by changing the weight of random sampling. For the high mobility scenario, all location adjustment values occurred with equal probabilities, corresponding to an overall probability of departing the present cell of 0.88 at each time step. For the low-mobility scenario, the overall probability of departing the present cell at each time step was 0.36.

### (b) Disease dynamics

Agents were initialized in a susceptible state, except for one randomly selected individual who was infected at the start of the simulation. Infection within groups was assumed to be driven by both direct and environmental transmission, whereas transmission between groups was assumed to be entirely environmental. The direct transmission was density dependent. Both direct and environmental transmission were stochastic and arose from Bernoulli processes. The force of infection for group i located at grid (*x*, *y*) is given as


$$\begin{array}{c} \lambda_{i}=\beta_{d}*I_{i}+\beta_{e}*E\left(x,y\right). \end{array}$$

Here, $\beta_{d}$ is the direct transmission rate, $I_{i}$ is the number of infected individuals in group i , $\beta_{e}$ is the transmission rate via environment and E(x,y) is the environmental risk dependent on pathogen persistence at grid location (x,y) . Grid cells became environmentally contaminated with probability 1 ( $E\left(x,y\right)=1$ ) when there was an infection within a group positioned at that cell owing to the shedding of pathogen propagules, representing a persistent environmental reservoir. The grid infection status is binary (1 for infected, 0 for not infected). If a susceptible group encountered the infected grid, infection could spread to the new group via environmental transmission. Environmental persistence was modelled as the time during which the infected grid remained infectious. Persistence time was deterministic and varied from short (*t* = 3 days) to long (*n* = 30 days). If an infected individual visited a contaminated grid, the persistence time ‘clock’ was reset. We further considered two different disease types: a short infection with a mean infectious period of 10 days, and a long infection with a mean infectious period of 30 days while keeping the transmission rates constant across simulations.

### (c) Variable importance of simulation parameters

The parameter importance of mobility, persistence, group size and infectious period was estimated for each measurement of the epidemic peak, time to peak, final epidemic size and the environmental contribution to the final epidemic size using the randomForest package in R using default parameters [37]. Briefly, for each parameter combination, we split each simulation block into 500 different test and training sets by bootstrapping. We then built trees from each bootstrapped dataset by comparing two predictors (mobility, persistence, group size, infectious period) at each split. Variable importance was calculated using the importance function inthe randomForest package and reported as the importance based on the increase in node purity. All simulations and parameter rankings were performed in R software (v. 4.2.2) [38].

## 3. Results

### (a) Short infectious period led to smaller, faster epidemics

**Figure 2.**
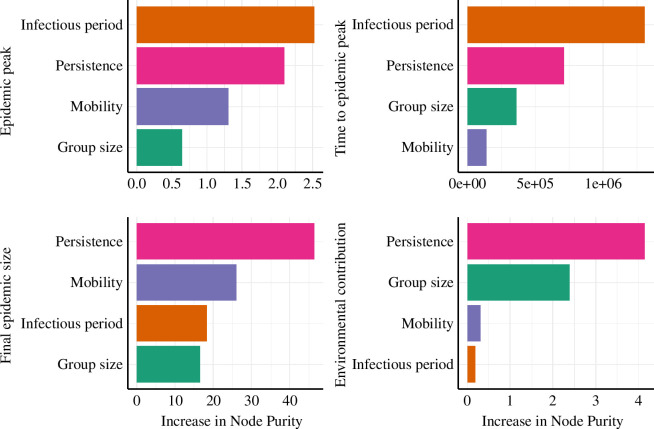
Variable importance ranking using the randomForest model. Model outputs from all scenarios were used to calculate variable importance. Simulation outputs using the torus lattice edge and scenarios with constant population size were excluded in calculating variable importance.

Diseases with short infectious periods led to lower epidemic peaks than those with longer infectious periods (electronic supplementary material, figures S1 and S2), and the latter reached their peak epidemic sizes more slowly, and with more heterogeneous timing (electronic supplementary material, figures S3 and S4). Epidemic peaks increased with group size, host mobility and environmental persistence. Within a disease type (short versus long infectious periods), larger social groups tended to have larger outbreaks, especially when combined with high mobility and longer environmental pathogen persistence (electronic supplementary material, figure S1, top left panel). Similar patterns were observed when dynamics occurred on a torus (electronic supplementary material, figure S5) and when the population size was kept constant instead of the number of groups (electronic supplementary material, figure S6). For diseases with long infectious periods, larger group sizes were associated with higher epidemic peaks, irrespective of group mobility and pathogen persistence (electronic supplementary material, figure S2). Group mobility had no effect on epidemic peaks for diseases when the infectious period and environmental persistence were short (electronic supplementary material, figure S1, right panels). However, higher group mobility led to larger epidemic peaks when pathogens had long environmental persistence, regardless of the infectious period (electronic supplementary material, figures S1 and S2). In general, the longer the environmental persistence of the pathogen, the higher the epidemic peak (table 2). The infectious period had the highest variable importance for explaining the epidemic peak, followed by persistence, mobility and group size (figure 2).

**Table 2.** Statistical comparisons for infectious period, pathogen persistence, group size and group mobility using Wilcoxon rank sum tests with correction for multiple testing ( α=0.01 ).

comparisons	epidemic peak	time to peak	final epidemic size	relative contribution of environmental infection
short infectious period (S) versus long infectious period (L)	L>S	L>S	L>S	L>S
short (S) versus long (L) persistence	L>S	L>S	L>S	L>S
mobility low (L) versus high (H)	H>L	H>L	H>L	H<L
group size: small (S) versus medium (M) versus large (L)	L>M>S	L>M>S	L>M>S	L>M>S

### (b) Larger group sizes and higher host mobilities resulted in longer time to peak prevalence

Group mobility and group size had little effect on time to peak prevalence (measured as time to epidemic peak) when the infectious period and environmental persistence were short (electronic supplementary material, figures S3 and S7, right panels). This was primarily owing to infection failing to move beyond the group where the infection was initiated (i.e. fade-out). A faster growth rate in these cases meant that the infection spread rapidly within groups but failed to spread between groups (electronic supplementary material, figures S1 and S3). For diseases with long infectious periods, the time to epidemic peak was more variable when groups were large (electronic supplementary material, figure S4). In general, the time to peak was longer for larger group sizes (table 2). Large variability in time to peak in most scenarios highlights the stochastic nature of transmission and group mobility. The infectious period had the highest variable importance for describing the time to epidemic peak, followed by environmental persistence, group size and mobility (figure 2).

### (c) Environmental persistence had a high influence on the final epidemic size

The cumulative proportion of the total population that was infected (the ‘final epidemic size’) at the end of the simulation shows how well infection spreads between groups. Diseases with short infectious periods and short environmental persistence only spread within the group where the infection was seeded and rarely transmitted to other susceptible groups (electronic supplementary material, figures S8–S10, right panels). On the other hand, a wide variability in the final epidemic size was observed for diseases with long infectious periods in host configurations of medium to high group sizes (electronic supplementary material, figure S4, right panel). Group size and mobility were positively associated with the final epidemic size when environmental persistence was long, regardless of the infectious period length (electronic supplementary material, figures S5, S8 and S9). When population size was kept constant for a disease with a short infectious period and long environmental persistence, wide variability was observed in the final epidemic size (electronic supplementary material, figure S6). Environmental persistence had the highest variable importance for describing the final epidemic size, followed by mobility, infectious period and group size (figure 2).

### (d) Relative contribution of infections via environment to total epidemic size

For diseases with short infectious periods, the longer the environmental persistence of pathogens, the greater the contribution of environmental transmission to the final epidemic size in medium- and large-group populations (electronic supplementary material, figures S11 and S12). A similar trend was observed in diseases with long infectious periods, but it was inclusive of smaller group sizes as well (electronic supplementary material, figure S13). The environmental contribution to the final epidemic size was negatively associated with the group size, especially for pathogens with long environmental persistence (electronic supplementary material, figures S11–S13, left panels). Environmental transmission accounted for a larger portion of transmission when mobility was lower and hosts were more localized. Environmental persistence had the highest variable importance for explaining the environmental contribution to transmission, followed by group size, mobility and infectious period (figure 2).

## 4. Discussion

This study offers insights into how group mobility, group size, the environmental persistence of pathogens and the infectious period interact to shape the epidemiology of wildlife diseases. Consistent with intuition, we show that long infectious period, high group mobility, large group size and long pathogen persistence all generate more intense outbreaks in terms of epidemic peaks observed in populations. We further show how these factors interact to either enhance or reduce the intensity of outbreaks. For example, mobility and persistence interact differently in diseases with short infectious periods depending upon pathogen persistence, which leads to different degrees of infection in the total population and relative contribution to total infection via the environment. Long persistence positively interacts with high mobility to increase the final epidemic size, but at the same time, high mobility and short persistence negatively interact to reduce the final epidemic size in populations. Thus, the intensity and direction of interactions change depending on the factors considered. This highlights the importance of considering spatial and social processes together while understanding disease dynamics and planning effective management actions [39].

The cost of increased disease transmission with group living has long been realized in social animals [40]. Increased infection prevalence is thought to be associated with larger group sizes and is supported to some extent by empirical findings (e.g. malaria in primates and brucellosis in elk; [41–43]). Consistent with this, we show that in diseases with short infectious periods, larger groups with high mobility experience larger epidemics, particularly when pathogens persist longer in the environment. However, the patterns observed for diseases with long infectious periods are much more nuanced and depend on interactions between group mobility and pathogen persistence. The longer infectious period allows the pathogen to persist longer within groups, allowing more spread to other groups [44]. Additionally, the environmental contribution to the final epidemic size shows a negative correlation with group size, particularly for pathogens with longer environmental persistence, a pattern that was consistent even when the population size was fixed. Longer environmental persistence and higher mobility increase the probability of exposure to more susceptible groups leading to infection seeding in many groups. The infection then rapidly spreads within groups with major contributions from direct transmission, thus leading to greater cumulative infection in populations with large group sizes. Group size also had an effect on the time it takes to reach epidemic peaks with populations with larger groups taking more time. This is simply because there are more susceptible individuals available in populations with larger groups that can sustain longer transmission chains compared with populations with smaller groups. Thus, management that targets the reduction of at least a proportion of susceptible individuals in social groups (e.g. vaccination by oral baiting targeted at large groups) could be an effective strategy under these conditions.

The role of host mobility in disease transmission is multifaceted, with varying impacts based on pathogen environmental persistence. In general, higher mobility led to larger epidemic peaks, however, the contribution to the final epidemic size via the environment was lower compared with the population with low group mobility. Highly mobile hosts can escape infected patches thereby reducing infection via the environment [45]. With short environmental persistence and shorter infectious period, infected patches can become pathogen free before a susceptible group encounters the patch thereby limiting intergroup transmission. Conversely, mobility has a positive effect on intergroup transmission if the number of groups is high enough such that the probability of between-group contact is high enough that it occurs before pathogens decay in the environment or before an infected host recovers. However, for both disease types with long and short infectious periods involving pathogens with long environmental persistence, increased mobility was associated with a higher proportion of the population becoming infected. This is akin to the spread of bovine tuberculosis in African buffalo, where greater mobility facilitates broader disease dissemination across the landscape [46]. Similar to our results, long environmental persistence can help sustain infection even in small populations [47].

Considering the variability in disease dynamics observed across different scenarios, no single management strategy will be universally effective. Adaptive management strategies that are responsive to local ecological and social factors are essential. For diseases caused by pathogens with short environmental persistence, our findings suggest that controlling movement between groups could be effective at reducing the spread, especially for diseases with a short infectious period [48,49]. Restricting the movement of owned free-ranging domestic dogs (via confinement) could be a way to control rabies transmission between groups of dogs [50]. Likewise, fencing can be effective in restricting the movement of infected groups to reduce the transmission and likelihood of the geographical spread of African swine fever in wild boar [51–53]. By contrast, for pathogens with longer environmental persistence, management strategies should extend beyond movement control. Here, environmental decontamination becomes crucial [54–56]. Decontamination (e.g. using chlorine dioxide against *Pseudogymnoascus destructans* [57]) can be targeted to areas based on the highest animal space use, using habitat selection inferences (see [58] for habitat selection-based targeting of a directly transmitted pathogen). Larger group sizes, particularly with long infectious periods, require strategies that address both the high rate of intra-group transmission and the potential for inter-group spread. Vaccination strategies might be more effective in these settings, as they can reduce transmission within large groups and prevent spillover to other groups [59]. Vaccination of wildlife has been successfully used in small populations of conservation concern (e.g. rabies in Ethiopian wolves; [60,61]) or in systems with established methods of mass deployment (e.g. raccoon rabies oral baits by hand-baiting, bait stations or aircraft; [62–64]). For diseases with short infectious periods in smaller groups, early detection via environmental surveillance [65] and quarantine might be more appropriate, given the limited spread of these infections. Thus, accounting for spatial and social processes along with the infectious period can be useful for developing targeted management strategies.

Although we uncover general patterns in disease dynamics based on social, spatial and environmental factors, our approach has several limitations. We considered a simple SIR dynamic without demographic turnover, and to incorporate multiple factors, we simplified host social and spatial aspects. Although we varied group sizes, we considered each group to be comprised of homogeneous individuals. Individual [66] and group [67,68] heterogeneities are known to influence transmission dynamics. Further, we considered a simple movement rule. However, animals show complex movement behaviour dependent on internal and external factors. Incorporating more realistic movement rules into disease models will help improve predictive power in wildlife systems [14,21,69]. Similarly, we considered a homogeneous landscape. However, spatial heterogeneity can shape movement patterns [70]. Additionally, pathogen environmental persistence can also be influenced by landscape features. High habitat quality areas that also permit the persistence of pathogens in the environment can lead to ecological traps and disease hotspots [71,72]. Although adding these features into the model can increase computational complexity, the introduction of more detailed spatial features can be suitable for understanding the system or locale-specific epidemiological dynamics and intervention strategies. Thus, our model provides a simplified framework for the further exploration of the complex interplay between social, spatial and environmental processes in disease dynamics.

The interplay between social and spatial processes in shaping animal communities has long been realized in ecology [39]. Infectious disease dynamics is similarly influenced, directly or indirectly, by these processes. Our study emphasizes the importance of host behaviour, particularly mobility and gregariousness, along with environmental factors, in disease transmission within wildlife populations. Previous research has provided valuable insights into how these individual processes can impact disease dynamics. Our work extends this by exploring the simultaneous interaction of these processes in determining disease outcomes. By considering these complex interactions, our study emphasizes how a nuanced understanding of disease ecology is important for refining disease management and control strategies across diverse animal populations. Crucially, this research highlights the need for integrated, adaptive management strategies tailored to specific ecological contexts and disease characteristics and lays a foundation for enhancing the context-specific understanding of disease dynamics.

## Data Availability

The code used in this manuscript is available at Zenodo [73]. Supplementary material is available online [74].
